# Clinical practice and implications of biomarker testing in biliary tract cancer: An observational study^☆^

**DOI:** 10.1016/j.jhepr.2025.101635

**Published:** 2025-11-04

**Authors:** Sabrina Welland, Ann-Kristin Zöller, Ilektra A. Mavroeidi, Aurelie Tomczak, Christian Müller, Dong Yawen, Danmei Zhang, Felix Keil, Maria Pangerl, Taotao Zhou, Hossein Taghizadeh, Sebastian Lange, Maximilian N. Kinzler, Kataryna Shmanko, Maryam Barsch, Carolin Zimpel, Angela Djanani, Henning Schulze-Bergkamen, Julius Keyl, Florian Lüke, Thomas Wirth, Michael Dill, Thomas Longerich, Sophia Petschnak, Jens U. Marquardt, Michael Quante, Arndt Weinmann, Dirk Walter, Nicole Pfarr, Gerald Prager, Bernhard Doleschal, Maria A. Gonzalez-Carmona, Rainer Günther, Alexander Scheiter, Stefan Böck, Stephan Bartels, Thomas Gruenberger, Marino Venerito, Christoph Springfeld, Stefan Kasper, Anna Saborowski, Arndt Vogel

**Affiliations:** 1 Dept. of Gastroenterology, Hepatology, Infectious Diseases and Endocrinology, Hannover Medical School, Hannover, Germany; 2 Department of Medical Oncology, West German Cancer Center, University Hospital Essen, Essen, Germany; 3 Institute of Pathology, Heidelberg University Hospital, Heidelberg, Germany; 4 Department of Gastroenterology, Hepatology and Infectious Diseases, Otto-von-Guericke-University Hospital Magdeburg, Magdeburg, Germany; 5 Department of Surgery, HPB Center Vienna Health Network, Clinic Favoriten and Sigmund Freud Private University, Vienna, Austria; 6 Department of Internal Medicine III, Grosshadern University Hospital, Ludwig-Maximilians-University, 81377 Munich, Germany; 7 Institute of Pathology, University of Regensburg, Regensburg, Germany; 8 Division of Hepatology, Department of Internal Medicine I, University Medical Center Schleswig-Holstein, Campus Kiel, Kiel, Germany; 9 Department of Internal Medicine 1, University Hospital of Bonn, Bonn, Germany; 10 Department of Internal Medicine 2, Gastroenterology and Hepatology and Rheumatology, Karl Landsteiner University of Health Sciences, University Hospital of St. Pölten, St. Pölten, Austria; 11 Technical University of Munich, School of Medicine and Health, Clinical Department for Internal Medicine II, TUM University Hospital, Munich, Germany; 12 Goethe University Frankfurt, University Hospital, Medical Clinic 1, Frankfurt am Main, Germany; 13 Department of Medicine I, University Medical Center Johannes Gutenberg University, Mainz, Germany; 14 Clinic for Internal Medicine II, University Medical Center Freiburg, Freiburg, Germany; 15 Department of Medicine I, University Hospital Schleswig-Holstein, Lübeck, Germany; 16 University Hospital of Innsbruck, Department of Gastroenterology, Hepatology and Endocrinology, Innsbruck, Austria; 17 Clinic for Gastroenterology, Hematology, Oncology, Diabetology, and Rheumatology, Marien-Hospital Wesel, Wesel, Germany; 18 Institute for Pathology, West German Cancer Center, University Hospital Essen, Essen, Germany; 19 Department of Internal Medicine III, Hematology and Oncology, University Hospital Regensburg, Regensburg, Germany; 20 Department of Gastroenterology, Infectious Diseases and Intoxication, Heidelberg University Hospital, Heidelberg, Germany; 21 Institute of Pathology, School of Medicine and Health, Technical University Munich, Munich, Germany; 22 Department of Internal Medicine I for Hematology with Stem Cell Transplantation, Hemostaseology, and Medical Oncology, Ordensklinikum Linz - Barmherzige Schwestern Site, Linz, Austria; 23 Medical Faculty, Johannes Kepler University Linz, Linz, Austria; 24 Department of Hematology and Oncology, München Klinik Neuperlach, 81737 Munich, Germany; 25 Institute of Pathology, Hannover Medical School, Hannover, Germany; 26 Department of Medical Oncology, Heidelberg University Hospital, Heidelberg, Germany; 27 Liver Cancer Centre Heidelberg, Heidelberg, Germany; 28 UHN Division of Hepatology, Toronto General Hospital, Toronto, Canada; 29 Division of Gastrointestinal Oncology, Princess Margeret Cancer Center, Toronto, Canada; 30 Institute of Clinical Pathology, Molecular Pathology and Microbiology, Vienna Health Network, Clinic Favoriten, Vienna, Austria

**Keywords:** panel sequencing, molecular targeted therapy, precision oncology, next-generation sequencing, biopsy

## Abstract

**Background & Aims:**

Biliary tract cancers (BTC) are aggressive malignancies with limited treatment options. Owing to the high frequency of actionable genomic alterations (GA) and the availability of targeted therapies, molecular testing has become increasingly important; however, its clinical implementation remains inconsistent. This study aimed to evaluate real-world molecular testing practices, characterize the BTC molecular landscape, and assess the prognostic and predictive relevance of selected GA.

**Methods:**

We retrospectively analyzed genomic and clinical data from 1,521 patients treated at 18 centers in Germany and Austria. A side-by-side comparison of clinical grade reports generated on two different sequencing platforms was performed for 90 patients.

**Results:**

Twenty-four different NGS panels were used across 18 centers. A comparative analysis highlighted the significant variability in reports used to inform therapeutic decisions in clinical practice. Although there were substantial differences in the number of GA covered, the broader panels identified a similar number of actionable GA, indicating that key therapeutic targets are sufficiently represented. Integration with clinical data suggested that certain GA, such as *HER2* amplifications (3%)*, BRAF*^*V600E*^ mutations (2%), and *FGFR2* alterations (14%), may have prognostic significance beyond their predictive value. Patients with actionable alterations (610, 40%) that were treated accordingly (n = 204, 13%) had prolonged overall survival (31.8 months *vs.* 22.8 months, *p* <0.01).

**Conclusion:**

Standardized biomarker testing is crucial for effective integration of targeted therapies in the management of BTC. Our findings reinforce the value of targeted treatments and underscore the predictive and prognostic significance of selected GA.

**Impact and implications:**

Genomic profiling is recommended in patients with biliary tract cancers (BTC) but lacks harmonization across platforms and centers. By retrospectively analyzing genomic and clinical information from 1,521 patients with BTC diagnosed and treated at 18 centers in Germany and Austria, we provide real-world insights into the implementation of molecular profiling in BTC, highlighting variability in next generation sequencing-based testing and its impact on the detection of genomic alterations. Standardized molecular testing strategies will be key to enable the integration of more consistent and comparable genomic datasets across studies. Further, by elucidating the prognostic relevance of individual genomic alterations, our insights carry significant implications for interpreting single-arm clinical trials within genomically stratified patient cohorts and underscore the importance of randomized studies to delineate the benefit of targeted therapies.

## Highlights

• Multiple NGS panels are used to assess actionable alterations in cholangioarcinoma.
• Detection rates of genomic variants vary by center and platform.
• Key actionable alterations were consistently reported.
• *BRAF*^*V600E*^ and copy number variations in *ERBB2* and *MTAP/CDKN2A/B* may be associated with poor survival.
• Targeted treatment was associated with improved outcomes.

## Graphical abstract

**Figure:**
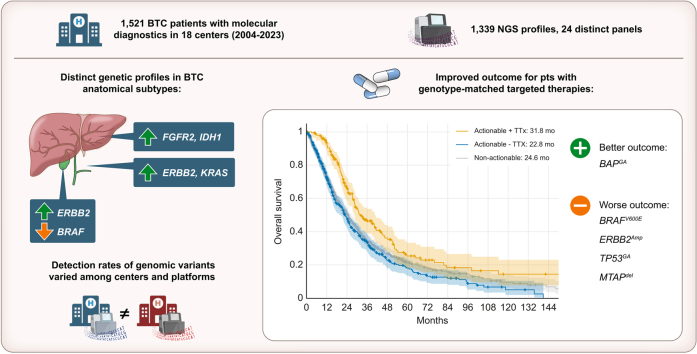

## Introduction

Biliary tract cancers (BTC) comprise a heterogeneous and highly aggressive group of malignancies arising in association with the biliary tree. Despite the relatively low incidence of less than 6 per 100,000 individuals worldwide, BTC remains a significant oncological challenge owing to the late onset of symptoms, complex diagnosis, and limited treatment options.^1^^,^^2^ In Europe, BTC predominantly affects individuals aged >60 years, with a slight male predominance. While most cases occur sporadically, several well-established risk factors contribute to disease development, including advanced age, metabolic dysfunction-associated steatotic liver disease, chronic viral hepatitis B and C infections, and primary sclerosing cholangitis.^3^^,^^4^

BTC is classified based on its anatomical location into intrahepatic cholangiocarcinoma (iCCA), extrahepatic cholangiocarcinoma (eCCA), and gallbladder carcinoma (GBC). iCCA are further subdivided into small and large duct subtypes, each with distinct histopathological and molecular characteristics, probably reflecting different cells of origin.^5^ Small duct iCCA frequently harbors *IDH1*, *IDH2*, and *BRAF* mutations, as well as *FGFR2* fusions, whereas large duct iCCA – similar to eCCA – more commonly harbors *KRAS* and *TP53* mutations. These genomic variations not only reflect the biological diversity of BTC but also underscore the potential for targeted therapeutic interventions.

Historically, systemic treatment options for BTC have been limited, with conventional chemotherapy providing only modest benefits.^6^^,^^7^ More recently, checkpoint inhibitors have been integrated into first-line therapy in combination with conventional chemotherapy, but their long-term benefits remain limited.[8], [9], [10] Advances in next-generation sequencing (NGS) have contributed significantly to comprehensive biomarker testing of the genomic landscape^11^^,^^12^ and have revealed that approximately 40% of patients with BTC harbor actionable genomic alterations (GA), paving the way for precision oncology strategies. Consequently, molecular testing has been increasingly recognized as a critical component of BTC management, enabling the identification of targeted therapies tailored to individual tumor profiles.

Recognizing the growing importance of biomarker testing, international guidelines, such as those published by the European Society for Medical Oncology (ESMO) or the European Association for the Study of the Liver, recommend multigene NGS panels for patients with BTC and the use of targeted therapies, particularly in those with level I actionable alterations^1^^,^^13^. They further emphasized the role of molecular testing in routine clinical practice to guide therapeutic decision-making. However, despite its advantages over single-gene testing, the routine integration of NGS into clinical workflows remains a challenge.

Beyond technical hurdles, such as insufficient tumor cellularity and/or poor quality of tissue samples, which can compromise the success of molecular testing, disparities in healthcare infrastructure, reimbursement policies, clinician awareness, and expertise influence the success and accessibility of NGS-based diagnostics^14^^,^^15^. The degree of investment in NGS infrastructure and its incorporation into routine clinical practice varies significantly, with no unified consensus on the best practices for NGS testing in BTC. Addressing these challenges is crucial to ensure equitable access to molecular diagnostics and expand personalized treatment opportunities for patients with BTC.

In this study, we aim to provide a comprehensive overview of the implementation of molecular testing for BTC across 18 centers in Germany and Austria. We present a detailed analysis of NGS panel use and its impact on the detection frequency of GA. Furthermore, we examine the predictive and prognostic significance of key GA in BTC, contributing to a better understanding of how biomarker testing can shape clinical decision-making and improve patient outcomes.

## Patients and methods

### Cohort description

The study population comprised patients diagnosed with BTC, mixed hepatocellular carcinoma/cholangiocarcinoma, and ampullary cancer.

Patients received treatment at 18 clinical institutions across Germany and Austria according to local recommendations. The data cut-off was 01.12.2024. Data were retrospectively extracted from genomic analysis reports. A total of 1,175 patients underwent molecular testing as part of clinical care, and 162 cases were sequenced retrospectively for research purposes.

Genomic alterations were assessed locally . In our retrospective analysis, we included only variants classified as pathogenic or likely pathogenic, either according to the reports provided by the commercial tests or as determined by the participating institutions in accordance with their local analysis standards. Actionable GA were classified according to the ESCAT criteria as recommended by the ESMO guidelines (19), and tier I, II, and III GA were considered actionable (listed in Table S3).

### Statistical analysis

Statistical analyses were conducted using IBM SPSS Statistics software, version 28 (SPSS Inc., Chicago, IL, USA), and lifeline survival analysis in Python 0.27.8. Patient survival was calculated from the date of initial diagnosis and the start of systemic treatment to the date of death or last follow-up, whichever occurred first. Categorical variables were examined using the chi-square test or Fisher's exact test, depending on their suitability based on the expected frequencies. Overall survival (OS) rates were estimated using the Kaplan-Meier estimator and differences between survival curves were assessed using log-rank tests. A *p* value of less than 0.05 was considered as the threshold for statistical significance in all analyses.

## Results

### Patient demographics

The retrospective cohort encompassed a total of 1,521 patients diagnosed with BTC who underwent molecular diagnostics in 18 gastrointestinal oncology centers in Germany and Austria between 2004 and 2023. The median age of the cohort was 64 years; the cohort was well balanced with respect to sex, and the majority of patients had iCCA (65.0%) (Table 1).

**Table 1:** Baseline characteristics of the full cohort (N = 1,521) and patients with panel sequencing (n = 1,339).

	Overall cohort (%) (N = 1,521)	Panel diagnostic (%) (n = 1,339)
Age (years)		
Median; range	64; 22-92	64; 22-92
Sex		
Male	789 (51.9)	694 (51.8)
Female	729 (47.9)	642 (47.9)
Missing	3 (0.2)	3 (0.2)
Status at last follow-up		
Dead	947 (62.3)	865 (64.6)
Alive	507 (33.3)	422 (31.6)
Lost to follow-up	67 (4.4)	52 (3.8)
Diagnosis		
iCCA	989 (65.0)	906 (67.7)
pCCA	245 (16.1)	190 (14.2)
dCCA	99 (6.5)	80 (6.0)
AC	36 (2.4)	25 (1.9)
GBC	129 (8.5)	116 (8.7)
unknown	23 (1.6)	22 (1.6)
Stage at diagnosis		
Liver limited	823 (54.1)	709 (53.0)
Metastatic	657 (43.2)	592 (44.2)
Unknown	41 (2.7)	38 (2.8)
Histology		
Adenocarcinoma	1,441 (94.7)	1,273 (95.1)
Mixed HCC/CCA	29 (1.9)	23 (1.7)
Other/unknown	51 (3.4)	43 (3.2)
Differentiation		
G1	40 (2.6)	34 (2.5)
G2	704 (46.3)	623 (46.5)
G3	377 (24.8)	353 (26.4)
unknown	400 (26.3)	329 (24.5)
No. of lesions at diagnosis		
Unilocular <5 cm	297 (19.5)	257 (19.2)
Unilocular >5 cm	212 (13.9)	189 (14.1)
Multilocular	527 (34.6)	481 (35.9)
Unknown	485 (31.9)	412 (30.8)
Vascular infiltration at diagnosis		
No	672 (44.1)	552 (12.2)
Yes	410 (27.0)	374 (27.9)
Unknown	439 (28.9)	413 (30.8)
Lymph node metastasis at diagnosis		
No	613 (40.3)	525 (39.2)
Yes	689 (45.3)	618 (46.2)
Unknown	219 (14.4)	196 (14.6)
Distant metastasis at diagnosis		
No	860 (56.5)	748 (55.9)
Yes	589 (38.7)	531 (39.7)
Unknown	72 (4.7)	60 (4.5)
Treatment intention at diagnosis		
Curative	638 (41.9)	550 (41.1)
Palliative	767 (50.4)	675 (50.4)
Unknown	116 (7.6)	114 (8.5)
Treatment		
Resection	774 (50.9)	656 (49.0)
Status after resection		
R0	456 (58.9)	391 (69.2)
R1	167 (21.6)	146 (25.8)
R2	28 (3.6)	25 (4.4)
Rx	39 (5.0)	34 (6.0)
Unknown	84 (10.9)	60 (9.1)
Neoadjuvant treatment	62 (8.0)	53 (10.6)
Adjuvant treatment	363 (46.9)	317 (56.1)
Locoregional treatment		
Any locoregional treatment	212 (13.9)	186 (13.9)
Radiotherapy (liver)	146 (9.6)	138 (10.3)
Radiotherapy (extrahepatic metastasis)	77 (5.1)	
SIRT	91 (6.0)	80 (6.0)
Ablation	68 (4.5)	56 (4.2)
TACE	46 (3.0)	44 (3.3)
Other	26 (1.7)	
Palliative stage	1,311 (85.4)	1,167 (87.2)
No. of palliative systemic treatment lines (% of patients at palliative stage/% of whole cohort)		
Median/mean	2/1.7	2/1.8
≥1	1,146 (87.4/75.3)	1,043 (89.4/77.9)
≥2	623 (47.5/41.0)	568 (48.7/42.4)
≥3	315 (24.0/20.7)	298 (25.5/22.3)
≥4	92 (7.0/6.0)	90 (7.7/6.7)
≥5	41 (3.1/2.7)	39 (3.3/2.9)
BSC only	133 (10.1/8.7)	100 (8.6/7.5)
Missing	29 (2.2/1.9)	24 (2.1/1.8)
Targeted therapies	205 (15.6/13.5)	195 (12.7/14.6)

AC*,* ampullary cancer*;* BSC*,* best supportive care; CCA*,* cholangiocarcinoma; dCCA*,* distal cholangiocarcinoma*;* GBC*,* gallbladder carcinoma*;* HCC*,* hepatocellular carcinoma*;* iCCA*,* intrahepatic cholangiocarcinoma*;* pCCA*,* perihilar cholangiocarcinoma*;* SIRT*,* selective intra-arterial radiotherapy; TACE*,* trans-arterial chemoembolization.

At initial diagnosis, 54.1% of patients had liver-limited disease, and 50.9% underwent resection. As expected, nearly all BTC cases were classified as adenocarcinomas, with mostly moderate (G2) differentiation (46.3%). 75% of patients within the full cohort received at least one line of palliative systemic therapy, and the median number of systemic treatments was two (mean, 1.7; range, 1–8 treatment lines). Only 13.5% of the full cohort received targeted therapy; however, the limited availability of targeted treatments in earlier years must be considered.

### Biomarker testing

In total, 1,339 patients underwent NGS-based panel diagnostics. The median time from the first diagnosis of advanced disease stage until the first molecular analysis was 107 days for patients diagnosed before January 1^st^ 2021, and 37 days for those diagnosed thereafter (calculations based on available data for n = 749 and n = 317 patients, respectively; Fig. S1). This observation is in line with the increasing availability of targeted treatments and supports the increased recognition of the importance of expedited molecular diagnostics in patients with BTC.

Twenty-four different panels were used in the 18 centers, the most frequent being Foundation Medicine CDx, TSO 500 and Oncomine Comprehensive Assay v3. DNA panels were complemented by the Archer FusionPlex Core Solid Tumor panel in 71 cases and *FGFR2* fluorescent *in situ* hybridization analysis was conducted alongside the Oncomine Focus Assay in 49 cases. Additional microsatellite instability (MSI)/mismatch repair and HER2 testing was reported in 57 patients.

**Fig. 1:**
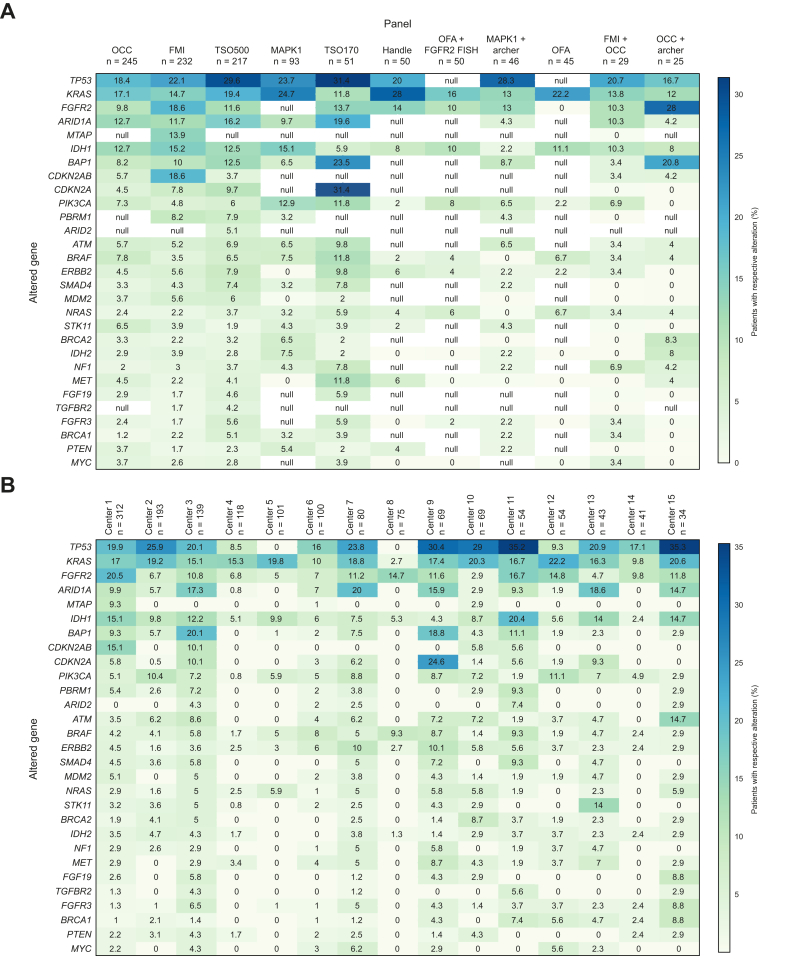
Percentage of patients with any alteration in specific genes separated by the applied NGS panel and the participating center. (A) Only included assays applied in at least 25 cases. Blanks indicate a lack of coverage, as per the technical specifications of the assay. (B) Depicts only participating centers that included more than 20 patients in the analysis. Zero indicates that no GA in the respective genes was reported, either because of a lack of coverage or non-detection in the respective cohort. Archer, Archer FusionPlex Core Solid Tumor Panel; FMI, FoundationOne CDx; GA, genomic alterations; Handle*,* HANDLE Classic NGS Panel; *MAPK1,* GeneRead DNA seq Custom Panel v2; NGS*,* next-generation sequencing; OCC, Oncomine Comprehensive Cancer Panel v3; OFA, Oncomine Focus Assay; *TSO170,* TruSight Oncology 170; *TSO500,* TruSight Oncology 500.

The coverage of the panels varied broadly, ranging from 15 to 523 genes, with distinct coverage of hotspots/genomic regions. With respect to the relative detection frequency of a specific GA (Fig. 1A), a very heterogeneous pattern emerged, although the small numbers must be considered in the interpretation of these results. As expected, *FGFR2* fusions were captured at different frequencies, likely due to the inability of some assays to detect rearrangements independently of defined partners. Similarly, most panels either did not cover or failed to detect deletions related to the *INK4A/ARF* locus spanning *CDKN2A* or *CDKN2A/B* with or without *MTAP*. Notably, the reported alteration frequency in known oncogenes, such as *KRAS*, and tumor suppressor genes, such as *TP53* *also varied considerably*.

The median number of reported GA per patient differed between the panels, ranging from 0.7–8.1 GA/patient (Table S1). However, a similar number of actionable GA per patient was detected by the larger panels, indicating that the most therapeutically relevant targets were sufficiently captured. Not surprisingly, the smaller panels yielded fewer total and/or actionable GA, highlighting the importance of extended biomarker testing for this disease. Similarly, the frequency of reported GA differed between centers, partly due to the sequencing panels employed (Fig. 1B).

### Comparative assessment of clinical reports generated on two different sequencing platforms

**Fig. 2:**
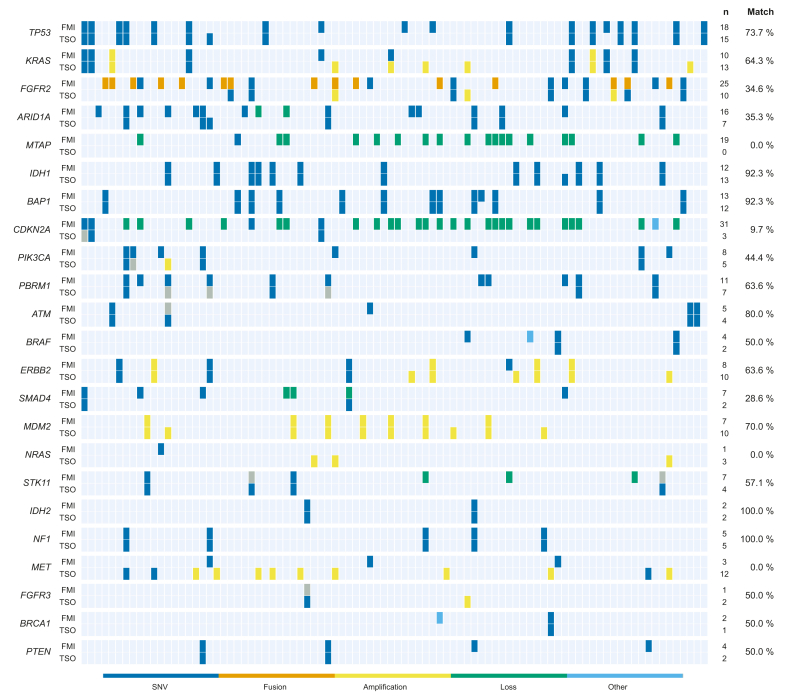
Direct comparison of the results of two independently generated reports for 90 patients sequenced on two different platforms (FMI CDx and TSO 500, DNA part). Each column represents an individual patient. Grey boxes indicate that the alteration was detected but was reported as a variant of uncertain significance. Only GA reported by at least one assay as clinically significant are shown. FMI*,* FoundationOne CDx; GA*,* genomic alterations; TSO*,* TruSight Oncology 500.

In view of the striking variability in the detection rates of specific GA, we generated and compared reports for 90 archived iCCA samples using two different panels (TSO 500 and Foundation CDx) (Fig. 2). Both sequencing and subsequent analyses were performed at two independent sequencing facilities. Of note, the TSO 500 included only the DNA part of the panel and not the RNA portion; thus, underreporting of *FGFR2* fusions was expected. Furthermore, the panel does not cover *MTAP* deletions, which may be of therapeutic relevance in the future, considering the emergence of targeted strategies for *MTAP*-deleted malignancies (PRMT5 inhibitors).^16^ Notably, *CDKN2A* deletions were not reported in the TSO 500 analysis, whereas amplifications, including those affecting *KRAS* and *MET*, were less frequent in the Foundation CDx report. With regard to single nucleotide variants, discordance also pertained to frequently known cancer genes, such as *TP53, PIK3CA* and *SMAD4*, amongst others. We acknowledge that the results may have been influenced by several potential confounding factors (further details in Table S2). Nevertheless, this side-by-side comparison highlights the degree of variability in reports that are used as the basis for therapeutic decision-making in clinical practice.

### Frequency of genomic alterations

**Fig. 3:**
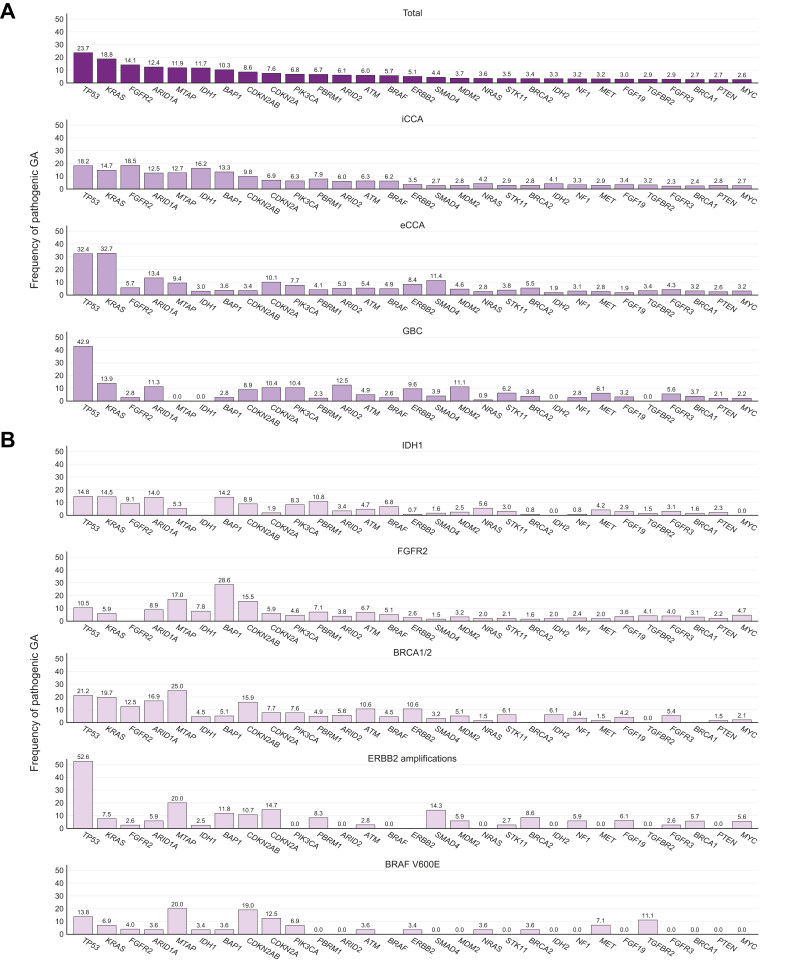
Frequency of GA. (A) Frequency of the respective GA in the full cohort and according to anatomic subtype and (B) co-alteration spectrum when stratified according to key actionable GA. Calculations were based only on panels that covered the respective GA. eCCA, extrahepatic cholangiocarcinoma; *FGFR2*, *FGFR2* fusions and activation mutations; GA, genomic alterations; GBCA, gallbladder carcinoma; iCCA, intrahepatic cholangiocarcinoma.

Overall, the reported GA and frequency with which they were reported were in line with previously published mutational profiles of biliary tract malignancies, most frequently affecting *TP53* and *KRAS* (Fig. 3A). Stratification according to the primary tumor site highlighted the higher rate of *FGFR2* and *IDH1* GA in iCCA than in GBC and eCCA, while *ERBB2* GA were more frequent in GBC and eCCA. eCCAs displayed the highest rate of *KRAS* mutations. *BRAF* GA were detected less frequently in GBC, whereas *BRCA1/*2 GA were observed at similar frequencies. Ampullary cancers were omitted from this analysis due to the overall low numbers (n = 25 with available panel diagnostics). Next, we focused on the co-alteration spectrum of the five key potentially actionable GA (Fig. 3B). Our real-world data confirmed previous reports regarding the high frequency of *TP5*3 GA and suggested an increased number of *SMAD4* co-alterations in *ERBB2*-amplified tumors. Mutational rates in *BAP1* stood out in *FGFR2*-altered malignancies while *TGFBR2* and *MET* GA were more frequent in *BRAF*^*V600E*^ tumors.

#### Therapeutic implications

Overall, 735 potentially actionable GA were identified across 610 patients, of whom 33% received targeted therapies (225 targeted treatments in 205 patients), amounting to approximately 13.5% of the full cohort (Table S4). The cohort included 29 patients diagnosed with mixed hepatocellular carcinoma/cholangiocarcinoma. Of the nine cases with actionable alterations, seven received targeted therapies, including four with *FGFR2* fusions and one each with a *FGFR2* mutation, an *ERBB2* amplification, and a *BRAF*^*V600E*^ mutation. The remaining two patients were found to have a *BRCA1* mutation and a *BRAF*^*V600E*^ GA, respectively, and did not receive targeted therapy. There was no statistically significant survival difference between patients diagnosed with adenocarcinoma *vs.* mixed tumors (*p* = 0.37).

**Fig. 4:**
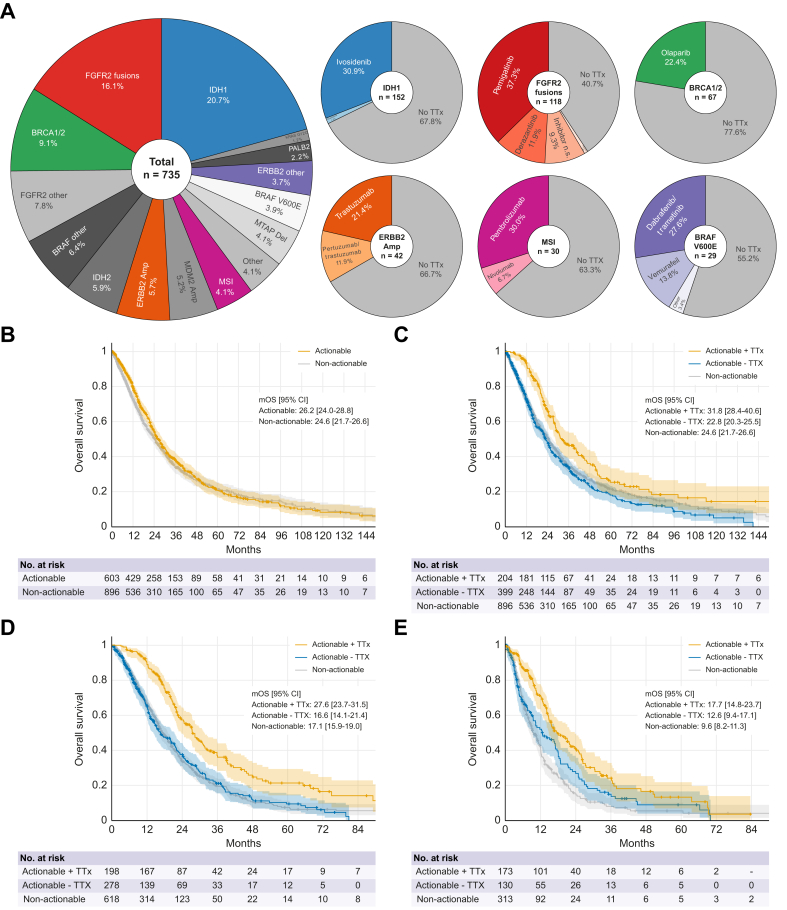
Actionable GA. (A) 735 actionable GA were identified in 610 patients (503 patients: one GA, 91 patients: two GA, 14 patients: three GA, 2 patients: four GA). Treatment allocation for six key actionable GA (small circles). For patients with sequential targeted therapies, only the first targeted treatment is shown (further specified in Table S1). (B) OS from diagnosis for patients with and without actionable GA (*p* = 0.207). (C) OS from diagnosis for patients with an actionable GA who received or did not receive targeted therapy, and for patients without actionable GA (+ TTx *vs.* - TTx: *p* <0.001; HR 0.62; 95% CI 0.50–0.77). (D) OS from start of palliative treatment (+ TTx *vs.* - TTx: *p* <0.001; HR 0.54; 95% CI 0.43–0.68 and (E) OS from start of second-line treatment (+ TTx *vs.* - TTx: *p* = 0.005; HR 0.66; 0.50–0.87). Amp, amplification; CTx*,* chemotherapy; Del, deletion; GA*,* genomic alteration; HR, hazard ratio, mOS, median overall survival; TTx, targeted therapy. Statistical tests: Kaplan-Meyer estimates, log-rank tests.

In the full cohort, FGFR and IDH1 inhibitors were the two most commonly used targeted therapies (Fig. 4A). Among patients with *FGFR2* fusions, 59.3% received at least one FGFR inhibitor. In contrast, only 30.9% of patients with an *IDH1* mutation were treated with ivosidenib. Although EMA approval for FGFR- and IDH1-targeted therapies was only granted in 2021 and 2023, respectively, relevant phase II and III trials were partially conducted during the study period and may have provided access to these investigational agents. In contrast, access to drugs targeting BRCA1/2, HER2, or BRAF^V600E^ was likely more challenging due to the lack of approval and/or ongoing clinical trials in Germany and Austria during the study period. Similarly, no immunotherapy had been officially approved and reimbursed for MSI-high tumors prior to 2023. Accordingly, only 36.7% of the patients with MSI-high tumors received pembrolizumab or nivolumab.

Next, we assessed the prognostic and predictive relevance of the selected GA. Overall, the presence of an actionable GA did not have a significant impact on the overall survival calculated from the first diagnosis in the full cohort (Fig. 4B). However, OS increased in patients who were treated with genotype-matched therapies compared to patients with actionable GA not receiving targeted treatments (Fig. 4C).

When calculated from the start of palliative systemic treatment, the OS of patients receiving targeted therapies was 27.6 compared to 16.6 months in patients with actionable GA not receiving targeted systemic therapies (Fig. 4D). This difference was maintained when survival was estimated from the start of second-line treatment (17.7 *vs.* 12.6 months) (Fig. 4E).

Notably, survival in our retrospective cohort exceeded survival reported in large clinical phase III trials. This observation likely reflects a selection bias, as our cohort also included patients in good physical condition who were referred to centers at later lines.

### Efficacy of genotype-matched therapies according to target alteration

**Fig. 5:**
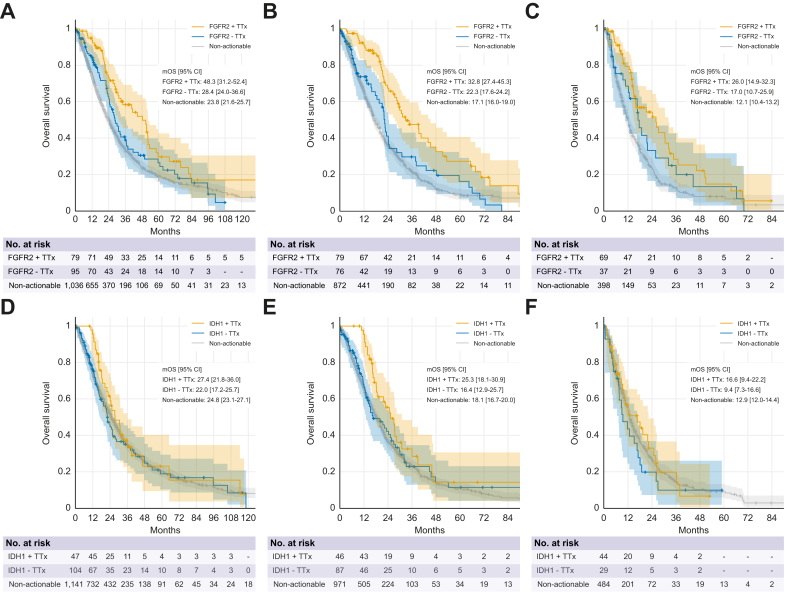
OS for patients with an actionable *FGFR2* GA (fusions and activating mutations) or *IDH1* mutations who received TTx, and for patients with and without the respective actionable GA. (A) OS for *FGFR2* GA from diagnosis (+ TTx *vs.* - TTx: *p* = 0.033; HR 0.65; 95% CI 0.45–0.96). (B) OS for *FGFR2* GA from the start of palliative treatment (+ TTx *vs.* - TTx: *p* = 0.001; HR 0.57; 95% CI 0.38–0.86). (C) OS for *FGFR2* GA from the start of second-line treatment (+ TTx *vs.* - TTx: *p* = 0.235; HR 0.72; 95% CI 0.44–1.19). (D) OS for patients with *IDH1* mutations from diagnosis (+ TTx *vs.* - TTx: *p* = 0.279; HR 0.81; 95% CI 0.53–1.24), (E) from start of palliative treatment (+ TTx *vs.* - TTx: *p* = 0.073, HR 0.71; 95% CI 0.45–1.11), (F) and from start of second-line treatment (+ TTx *vs.* - TTx: *p* = 0.310; HR 0.75; 95% CI 0.43–1.30). HR, hazard ratio; mOS, median overall survival; TTx, targeted therapy. Statistical tests: Kaplan-Meyer estimates, log-rank tests.

Next, we aimed to better delineate the predictive and potential prognostic relevance of the individual target GA. The efficacy of FGFR inhibitors has thus far been evaluated in single-arm phase II trials in second-line and randomized first-line studies that have recently been terminated due to poor accrual. Thus, while pemigatinib and futibatinib have received EMA and FDA approval, there is still an ongoing debate regarding the potential prognostic impact of *FGFR2* fusions. According to our real-world data, the median OS (mOS) of patients with *FGFR2* GA receiving inhibitor treatment exceeded 48 months from the first diagnosis and 32.8 months from the start of palliative systemic therapy, whereas the mOS in patients without targeted treatment was 28.4 and 22.3 months, respectively, thus further supporting their positive predictive value (Fig. 5A,B). When calculated from the start of second-line therapy, significance was lost, although a trend towards longer survival in patients undergoing targeted treatment remained (Fig. 5C). Nine patients with activating non‑fusion FGFR2 GA received targeted therapies. 47 patients from this cohort did not receive targeted treatment and were included in the OS analysis from diagnosis (Fig. 5A), and 37 patients were included in the analysis from the start of systemic therapy (Fig. 5B). The survival benefit of FGFR inhibitors was statistically less pronounced when the analysis was focused exclusively on patients with *FGFR2* fusions; however, the low number of *FGFR2* fusion-positive patients not receiving targeted treatments must be taken into consideration (Fig. S2A–C). Notably, the mOS in patients with *FGFR2* fusions who did not receive targeted treatment was 30 and 24.2 months from diagnosis and start of palliative therapy, respectively, which was longer than that of patients without *FGFR2* GA. This observation supports the assumption that *FGFR2* fusions have prognostic implications. In contrast, this pattern was not observed in patients with *IDH1* mutations, suggesting that the presence of *IDH1* mutations did not confer a survival advantage (Fig. 5C-E). In the pivotal ClarIDHy phase III trial, which established ivosidenib as the second-line treatment, the primary progression-free survival endpoint was reached, while there was no formal OS benefit.^17^ However, high crossover to the experimental arm must be considered. In our cohort, we observed a trend towards a longer mOS from the start of palliative therapy and the start of second-line therapy in patients with *IDH1* mutations under targeted treatment, compared to those not receiving targeted therapy, suggesting that a subset of patients benefited from treatment with ivosidenib (Fig. 5C-E).

**Fig. 6:**
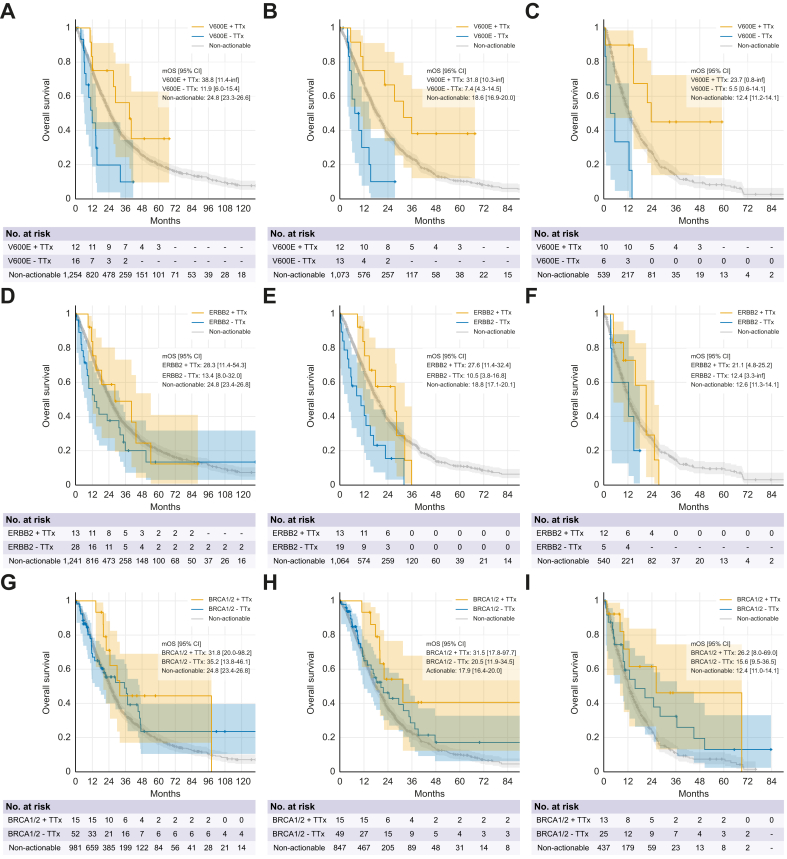
OS for patients with a *BRAF*^*V600E*^ mutation, *ERBB2* amplification or *BRCA1/2* alteration who received or did not receive TTx, and for patients without the respective GA. (A) OS for *BRAF*^*V600E*^ patients from diagnosis (+ TTx *vs.* - TTx *p* = 0.015; HR 0.27; 95% CI 0.11–0.69, (B) from the start of palliative treatment (+ TTx *vs.* - TTx: *p* = 0.003; HR 0.19; 95% CI 0.07–0.51), and (C) from the start of second-line treatment (+ TTx *vs.* – TTx: *p* <0.001; HR 0.12; 95% CI 0.04–0.38). (D) OS for *ERBB2* amplified patients from diagnosis (+ TTx *vs.* - TTx: *p* = 0.218; HR 0.64; 95% CI 0.29–1.38), (E) from the start of palliative treatment (+ TTx *vs.* - TTx: *p* = 0.012; HR 0.42; 95% CI 0.19–0.95), and (F) from the start of second-line treatment (+ TTx *vs.* - TTx: *p* = 0.113; HR 0.62; 95% CI 0.19–2.07). (G) OS for *BRCA1/*2 GA from diagnosis (+ TTx *vs.* - TTx: *p* = 0.340; HR 0.71; 95% CI 0.33–1.55), (H) from start of palliative treatment (+ TTx *vs.* - TTx: *p* = 0.179; HR 0.62; 95% CI 0.29–1.36) and (I) from the start of second-line treatment ((+ TTx *vs.* – TTx: *p* = 0.402; HR 0.68; 95% CI 0.27–1.72). GA, genomic alterations; HR, hazard ratio; mOS, median overall survival; TTx, targeted therapy. Statistical tests: Kaplan-Meyer estimates, log-rank tests.

As expected, the number of patients with *BRAF*^*V600E*^ mutations, *ERBB2* amplifications, or *BRCA1/2* mutations was low overall. Nevertheless, our retrospective data suggest a negative prognostic implication, especially when assessed from the start of palliative therapy, for both patients with *BRAF*^*V600E*^ or *ERBB2* amplifications. However, targeted treatment is also associated with survival benefits. The efficacy of BRAF-targeted therapies has been demonstrated in single-arm phase II basket studies and resulted in a tumor-agnostic FDA (but not EMA) approval for dabrafenib in combination with trametinib. Patients with *BRAF*^*V600E*^ mutations included in our retrospective analysis reached an mOS of 31.8 months from the start of treatment if they received targeted therapy, while the mOS was 7.4 months in those with *BRAF*^*V600E*^ mutations without targeted therapy and 18.6 months in non-*BRAF*^*V600E*^ patients. The benefit of targeted treatment remained when calculated from the start of second-line therapy (Fig. 6A-C). For *ERBB2*-amplified biliary cancers, multiple phase II trials have established efficacy for different HER2-targeted regimens, resulting in regulatory approval of the bi-paratopic antibody zanidatamab in biliary cancers and a tumor agnostic FDA approval for the antibody-drug conjugate trastuzumab-deruxtecan.^18^ The use of HER2-targeted agents resulted in an mOS of 27.6 months after the start of palliative treatment in our cohort, while the mOS of *ERBB2*-amplified patients not receiving targeted therapy was 10.5 months. Non-*ERBB2* amplified patients reached an mOS of 18.8 months. From the start of second-line treatment, a trend towards better survival under targeted therapy remained, although it failed to reach significance and the small patient numbers need to be considered (Fig. 6D-F).

To date, targeted therapies with PARP inhibitors have only been tested in small basket and retrospective trials, including patients with *BRCA1/2-*mutant biliary cancers.^19^^,^^20^ However, extrapolating from positive data on PARP inhibitor maintenance in pancreatic cancer, these agents might also be a valid option for BTC.^21^ *Post hoc* analyses from Topaz-1 and phase II basket trials suggested the superior efficacy of platinum agents with or without immunotherapy in a subgroup of *BRCA1/2* mutant patients.^22^ While our data do not suggest a clear prognostic or predictive implication for BRCA1/2, long-term survival appears to be possible in a subgroup with *BRCA1/2* mutations (Fig. 6G-I). Of note, our analysis does not consider germline *BRCA1/2* status (as in the POLO trial for pancreatic cancer) or homologous recombination deficiency, and thus we cannot distinguish bystander GA from GA with likely functional relevance.

**Fig. 7:**
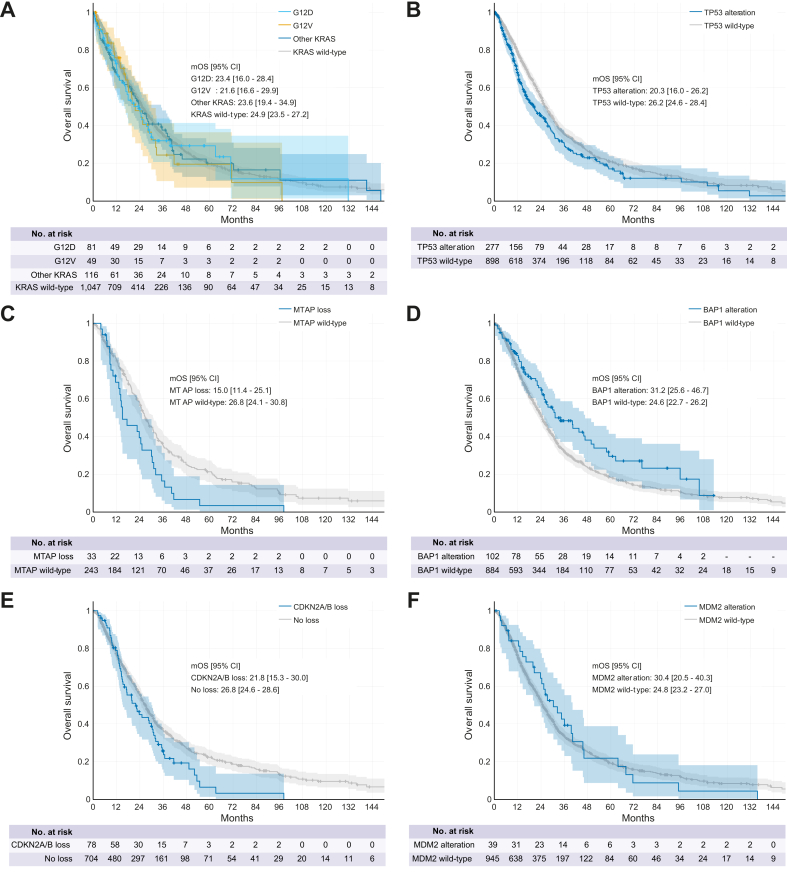
Overall survival from diagnosis for patients with and without specific GA. Overall survival from diagnosis for patients with and without GA in *KRAS* (A: *p* = 0.618), *TP53* (B: *p* = 0.003; HR 1.29; 95% CI 1.09–1.52), *MTAP* (C: *p* = 0.003; HR 1.76; 95% CI 1.2–2.6), *BAP1* (D: *p* = 0.008; HR 0.69; 95% CI 0.53–0.91), *CDKN2A/B* (E: *p* = 0.022; HR 1.36 1.1–1.8]), and *MDM2* (F: *p* = 0.819; HR 0.96; 95% CI 0.66–1.38). GA, genomic alterations; HR, hazard ratio; mOS, median overall survival. Statistical tests: Kaplan-Meyer estimates, log-rank tests.

Finally, we aimed to assess the potential prognostic implications of additional GA that were not considered actionable during the study period. In our cohort, GA in *KRAS* was not of prognostic relevance (Fig. 7A). However, considering ongoing trials, it is expected that in the near future, mutant KRAS will also become a key actionable target in BTC, beyond the fairly small subgroup of *KRAS*^*G12C*^ altered patients. In line with *post hoc* analyses from clinical trials, the presence of *TP5*3 GA appeared to be associated with shorter OS (20.3 *vs.* 26.2 months), as was *MTAP* loss (15.0 *vs.* 26.8 months) and *CDKN2A/B* deletion (21.8 *vs.* 26.8 months) (Fig. 7B-E). An inverse association was observed for patients with *BAP1* alterations, with an mOS of 31.2 *vs.* 24.6 months in the altered *vs.* non-altered cohort (Fig. 7D). Of note, considering that *BAP1* GA frequently co-occurs with *FGFR2* fusions, our observation of *BAP1* mutants and *FGFR2* fusion-positive BTC may be related. We did not observe a significant effect of *MDM2* GA on mOS (Fig. 7F).

## Discussion

Targeted therapies are rapidly evolving in BTC, opening new avenues of treatment for individual patients but also posing new challenges for healthcare providers. Here, we aimed to assess how molecular testing is performed in clinical practice, to interrogate the association between NGS panels and the detection rate of GA, and to delineate the prognostic and predictive implications of selected recurrent GA in BTC.

Our retrospective analysis included more patients with iCCA than eCCA, reflecting the distribution observed in recent phase III trials in an advanced setting. This predominance may be due to easier access to adequate tissue for molecular analysis in iCCA, as well as the higher prevalence of GA with EMA/FDA-approved targeted therapies, such as *FGFR2* fusions and *IDH1* mutations.

Our clinical cohort included not only patients who were diagnosed and received diagnostics between 2004 and 2023 but also molecular and clinical data from samples that were profiled for research purposes. In earlier years, NGS was not yet considered part of routine clinical workup, and no targeted treatments have been approved. Moreover, NGS was performed in the research cohort retrospectively; thus, NGS results did not qualify for targeted treatments. Collectively, these factors likely contributed to the relatively small number of patients who received targeted therapies, although similar limited uptake has also been reported in other studies.^23^ Although the extended duration of the study represents a limitation in terms of how reflective it is of the current treatment landscape, it provides an important opportunity to evaluate the prognostic significance of individual GA. This is especially important when interpreting survival data derived predominantly from single-arm phase II studies, which are often considered insufficient for universal approval by regulatory agencies. For instance, our data support the notion that *FGFR2* and *BAP1* GA often co-occur and are associated with a better prognosis. On the contrary, we provide evidence for a negative prognostic role of *BRAF*^*V600E*^ mutations analogous to colorectal cancer and for *ERBB2* amplifications, as reported for gastric cancer^24^^,^^25^ and small BTC cohorts.^26^ Thus, single-arm trials may tend to overestimate the efficacy of FGFR inhibitors while underestimating the benefits of BRAF- or HER2-targeted agents. Overall, we observed a survival benefit for patients with actionable alterations receiving targeted therapy. However, this benefit was less pronounced in the respective genomic subgroups, or when calculated from the start of second-line therapy. While we acknowledge the limitation of a retrospective analysis and the low patient numbers, these data support the necessity of randomized trials to truly assess the benefit of targeted therapies over conventional therapies.

Currently, biomarker testing is increasingly performed during the first line of systemic treatment, as recommended by the ESMO clinical practice guidelines. While we observed a significant shift towards earlier testing in the more recent patients included in our cohort, the majority were likely tested later. Therefore, molecular profiles might be enriched for positive prognostic GA, whereas, on the contrary, GA associated with a worse prognosis could be underrepresented.

We observed a notable heterogeneity in tests employed and results reported by the different centers. Our analysis, however, relies on routine clinical reports rather than raw sequencing reads or uniform pipelines. Tests differ in panel content, intronic coverage, and sequencing technologies used (*e.g.* RNA or DNA-based methods; amplicon *vs.* hybrid-capture approaches); interpretation of results, including copy number “amplification” calls and reporting thresholds also vary by laboratory. Because we do not have access to the original raw data, test-specific pipelines, or consistent metadata (*e.g*. tumor purity, bioinformatic thresholds), we cannot reprocess or harmonize results to adjudicate true differences *vs.* reporting artefacts.

Regarding our head-to-head comparison of retrospectively generated reports for 90 retrospective iCCA samples, we do not advocate for one testing platform over another. However, we want to emphasize that NGS results communicated to the healthcare provider are subject to inherent variability, influenced by technical prerequisites of the respective sequencing chemistries, subsequent analysis pipelines, as well as differential reporting and interpretation of primary data. Given that DNA was extracted from different section planes, intra-tumoral heterogeneity and clonal events may have further contributed to discrepancies in the results.

The direct clinical implications of our key findings are as follows: Although the cohort included only patients treated at gastrointestinal oncology centers, the mutational profiles differed with respect to the reported GA frequencies. These findings support the need for a more standardized approach to genomic profiling and reporting, consistent with the recent ESMO recommendations.^27^ As additional targeted therapies become available, it is critical to implement robust assay characteristics and reliable analysis pipelines to ensure that patients with BTC are not denied potential therapeutic options – in the near future, this may also extend to circulating tumor DNA-based testing, which may become especially useful in cases with limited access to tumor tissue, or for longitudinal testing under (targeted) therapy.

It must also be acknowledged that not all healthcare providers are nor should be expected to be experts in NGS-based diagnostics. This highlights the importance of providing well-annotated and user-friendly genomic reports. Moreover, our retrospective analysis offers important insights into the predictive and prognostic relevance of genomic alterations in BTC. These insights are essential not only for interpreting single-arm studies involving targeted therapies, but also for informing future trials that focus on genomic subgroups within this rare and genomically diverse cancer type.

## Abbreviations

BTC, biliary tract cancer; CCA, cholangiocarcinoma; eCCA, extrahepatic CCA; ESMO, European Society of Medical Oncology; GA, genomic alterations; GBC, gallbladder cancer; iCCA, intrahepatic CCA; NGS, next-generation sequencing; (m)OS, (median) overall survival.

## Financial support

This work was funded by the Deutsche Forschungsgemeinschaft (DFG, German Research Foundation) project numbers 493345156 (to ASa) and 348083549 (to AV). ASa was supported by the German Cancer Aid (70114101). AV was supported by the European-Latin American ESCALON consortium, funded by the EU Horizon 2020 program (project number 825510). ASa and AV are supported by the ERA-NET TRANSCAN-3 JTC22 consortium “PRECEDENCE.” This publication is based on work from COST Action CA22125, supported by COST (European Cooperation in Science and Technology). We thank the Molecular Tumor Board Freiburg (MTB-FR) Network (Center for Personalized Medicine, University Freiburg - Medical Center) for providing molecular and clinical data. JUM was supported by a grant from the Wilhelm-Sander Foundation. IAM was supported by the Clinician Scientist Program of the University Medicine Essen Clinician Scientist Academy (UMEA), Faculty of Medicine, and the Deutsche Forschungsgemeinschaft (DFG). Part of the work (panel sequencing of the retrospective iCCA cohort, MHH) was supported by a research grant provided by AstraZeneca (to ASa and AV).

## Authors’ contributions

Conceptualization: ASa, AV. Data curation: all authors. Formal analysis: ASa, AV, SW, AKZ. Investigation: all authors. Methodology: ASa, AV, SW, AKZ. Visualization: ASa, AV, SW, AKZ. Writing – original draft: ASa, AV. Writing – review & editing: all authors.

## Data availability

Data are available from the authors upon reasonable request.

## Ethics

The study was conducted in accordance with the Declaration of Helsinki, and the protocol was approved by the Ethics Committee of each institution involved in the project. This study was approved by the Ethics Committee of the Medical School Hannover (the principal investigator's institution). Ethical approval for this study was provided by the Ethical Committees of each participating institution: Hannover (3612-2017), Heidelberg (S-693/2019), Essen (21-10239-BO), Magdeburg (26/23), Munich (486/23), Regensburg (20-1682-101), Kiel (Broad consent of UK-SH), Bonn (341/17- V. 2.0), St. Pölten (1010/2025), Frankfurt (SGI-6-2024), Freiburg (24-1065-S1-retro), Lübeck (Broad consent and 21-448), Linz (1100/2023 and 1122/2024). Wesel, Mainz, Kiel, and Vienna: No formal ethics approval was required for this strictly retrospective study, as ruled by local ethics committees.

## Patient and public involvement

Patients and/or the public were not involved in the design, conduct, reporting, or dissemination of this research.

## Conflict of interests

AV reported personal fees from Roche, AstraZenca, Böhringer-Ingelheim, Ipsen, Incyte, Cogent, EISAI, Zymeworks, Biologix, BMS, Terumo, Elevar, Servier, MSD, Taiho, Jazzpharma, Medivir, Abbvie, Tyra, Janssen, and Lilly. ASa reports personal fees from BMS, Roche, Servier, Ipsen, Lilly, AstraZeneca, MSD, Eisai, Amgen, Taiho, Incyte, and Jazz Pharma, and travel support from Ipsen, Servier, Pierre-Fabre, MSD, and Eisai. AZ reports ownership of stocks in Novo-Nordisk and Vertex Pharmaceuticals. SK reports personal fees as speakers or consultants from BMS, Servier, Lilly, AstraZeneca, MSD, Taiho, Incyte, Daiichi Sankyo, Amgen, Oncowissen. de, and institutional funding from BMS, Roche, and Lilly. MV received personal fees from Servier, Roche, BMS, MSD, EISAI, Bayer, Lilly, AstraZeneca, Merck Serono, Sirtex, Ipsen, Incyte, Daichi-Sankyo, Böhringer Ingelheim, and Amgen and travel support from Servier, AstraZeneca, Amgen, and Ipsen. NP reports personal fees from Novartis, Eli Lilly, Roche, AstraZeneca, Johnson and Johnson, Bayer, Illumina, BMS, MSD, PGDx/LabCorp, GSK, and QuiP. ASc received travel support from Roche. SL has attended advisory boards or served as a speaker for Taiho, AstraZeneca, Janssen-Cilag, and MSD, and has received research funding from Illumina. JUM reports honoraria and travel support from AstraZeneca, EISAI, Taiho, Ipsen, MSD, ABBVIE, Janssen and Roche. AW received compensation as a member of the scientific advisory boards for AstraZeneca, Bayer, BMS, MSD, Eisai, Servier, and Sanofi. He served as a speaker for Leo Pharma, Eisai, Ipsen, Abbvie, AstraZeneca, and Roche, and received travel support from Merck and Servier. IAM received travel support from Pierre Fabre and speaker fees for Incyte. MQ has received honoraria/speakers” fees from Amgen, BMS, Celgene, MSD, Merck, Servier; served on advisory boards for Amgen, BMS, Incyte, MSD, Servier; has received travel support by Merk, Amgen. MB served on advisory boards for Taiho. MG has contributed to advisory boards for Roche, Eisai, MSD, BMS, AZ, Daiichi Sankyo, Amgen, and Servier, has received honoraria as speaker from BMS, AZ, Lilly, and MSD, and travel support from Servier, BMS, AZ, Lilly, and Amgen. DZ received honoraria from AstraZeneca, research funding from Milteny, and travel support from both AstraZeneca and Amgen. SP receives honoraria from/speakers’ fees from AstraZeneca, Servier, Stemline, Johnson&Johnson, Austrian Institute for Health Technology Assessment GmbH. SW, CM have no conflicts to declare.

Please refer to the accompanying ICMJE disclosure forms for further details.
