# Supplementary material for: Clinical practice and implications of biomarker testing in biliary tract cancer: An observational study
Source: JHEP Rep. 2025 Nov 4;8(1):101635. doi: 10.1016/j.jhepr.2025.101635 (PMC12890449; doi:10.1016/j.jhepr.2025.101635)
Supplement: Multimedia component 1 [file mmc1.pdf]

Clinical practice and implications of biomarker testing in biliary tract cancer: An observational study☆

Sabrina Welland, Ann-Kristin Zöller, Ilektra A. Mavroeidi, Aurelie Tomczak, Christian Müller, Dong Yawen, Danmei Zhang, Felix Keil, Maria Pangerl, Taotao Zhou, Hossein Taghizadeh, Sebastian Lange, Maximilian N. Kinzler, Kataryna Shmanko, Maryam Barsch, Carolin Zimpel, Angela Djanani, Henning Schulze-Bergkamen, Julius Keyl, Florian Lüke, Thomas Wirth, Michael Dill, Thomas Longerich, Sophia Petschnak, Jens U. Marquardt, Michael Quante, Arndt Weinmann, Dirk Walter, Nicole Pfarr, Gerald Prager, Bernhard Doleschal, Maria A. Gonzalez-Carmona, Rainer Günther, Alexander Scheiter, Stefan Böck, Stephan Bartels, Thomas Gruenberger, Marino Venerito, Christoph Springfeld, Stefan Kasper, Anna Saborowski, Arndt Vogel

Table of contents

Supplementary Materials and Methods……………………………………………….2

Table S1…………………………………………………………………………………..3

Table S2…………………………………………………………………………………..3

Table S3…………………………………………………………………………………..4

Table S4…………………………………………………………………………………..4

Fig. S1……………………………………………………………………………………6

Fig. S2……………………………………………………………………………………7

Supplementary Materials and Methods

Panels

Commercial assays included the Oncomine Comprehensive Assay v3, Oncomine Focus Assay, FoundationOne CDx (F1CDx), AmoyDx HANDLE Classic NGS Panel, Archer FUSIONPlex Core Solid Tumor Panel, Archer FUSIONPlex Lung v2 panel, TruSight Oncology 500, TruSight Tumor 170, TruSight Tumor 15, TruSight RNA Pan-Cancer Panel, QIAseq Targeted DNA Human Comprehensive Cancer Panel (Qiagen v3), QIAseq Targeted DNA Human Actionable Solid Tumor Panel, QIAseq Targeted DNA Human Tumor Mutational Burden Panel, custom Qiaseq panel for Netzwerk Genomische Medizin (nNGM v2), AmpliSeq Focus Panel, and AmpliSeq Cancer Hotspot panel. Additionally, custom panels were used at some sites, and selected GA were identified by focused testing, including BRCA (QiaSeq BRCAplus Panel, Oncomine BRCA Research Assay), immunohistochemistry (ERBB2, TRK A-C (Ventana-pan-TRK)), and fluorescence in situ hybridization (FGFR2).

Patient and public involvement statement

Patients were not involved in the design and execution of this study. Upon publication, the results of this real-world data analysis will be communicated to patient organizations and advocacy groups to support dissemination.

Table S1

Panel	No. Pat. ≥ 1 GA (n, %)	No. Pat. ≥ 1 druggable GA (n, %)	Druggabe GA per patient (n)	Median/mean GA per patient (n)
**OCC** n= 180	155 (86.1)	81 (45.0)	0.45	2.72/2.0
**OCC + Archer** n=25	23 (92.0)	12 (48.0)	0.48	2.40/2.0
**FMI** n= 232	190 (81.9)	130 (56.0)	0.56	3.75/3.0
**OFA** n=45	24 (53.3)	10 (22.2)	0.22	0.69/1.0
**OFA + FGFR2 FISH** n=50	29 (58.0)	14 (28.0)	0.28	0.67/1.0
**Handle** n=50	40 (80.0)	17 (34.0)	0.34	1.02/1.0
**MAPK1** n=93	79 (84.9)	40 (43.0)	0.43	1.76/2.0
**MAPK1+Archer** n=46	34 (73.9)	11 (23.9)	0.22	1.30/1.0
**TSO500** n=217	210 (96.8)	109 (50.2)	0.50	8.10/4.0
**TSO170** n=51	48 (94.1)	29 (56.9)	0.57	0.57/1.0

Table S1: Most frequently used NGS panels and detected (druggable) GA
OCC: Oncomine Comprehensive Cancer Panel (Thermo Fisher Scientific), Archer: Archer FusionPlex Core Solid Tumor panel (IDT), FMI: FoundationOne CDx (FoundationMedicine), OFP: Oncomine Focus Assay (Thermo Fisher Scientific), Handle: AmoyDx ® HANDLE Classic NGS Panel (Zytomed Systems), TSO 500: TruSight Oncology 500 High Throughput Panel (Illumina), TSO 170: TruSight Oncology 170 Panel (Illumina), MAPK1: GeneRead DNAseq Custom Panel V2 (Qiagen)
Potential actionable alterations are listed in Supplementary Table S3.

Table S2:

	Modifiers
Sample dependent	• intratumoral heterogeneity: isolation of DNA/RNA from different tumor areas/ section planes<br>• variable cellularity, scarce tissue<br>• sample quality
Sequencing dependent	• read depth, target genes and coverage<br>• filtering strategy<br>• variant annotation and reporting

Table S3:

Actionable alterations
• FGFR2 fusions (ESCAT I) and pathogenic SNV (ESCAT II)<br>• IDH1 (ESCAT I) and IDH2 hotspot mutations (ESCAT III)<br>• BRCA1 and BRCA2 alterations (ESCAT III)<br>• ERBB2 amplifications/overexpression (ESCAT I) and other pathogenic ERBB2 alterations (ESCAT II)<br>• BRAF V600E mutations (ESCAT I) and other pathogenic BRAF alterations (ESCAT III)<br>• MDM2 amplifications (ESCAT II)<br>• KRAS G12C mutation (ESCAT II)<br>• MET amplification (≥8-fold), MET exon 14 skipping (ESCAT III)<br>• MSI high (ESCAT I)<br>• MTAP deletion (ESCAT II)

Table S4:

	No. of actionable GA	TT start overall cohort	Treatment
Overall	**735** actionable GA in **610** patients	**225** actionable GA in **205** patients	
FGFR2 fusion	118	70 patients	
		1 FGFRi:<br>62 patients	Pemigatinib n= 40<br>Derazantinib n=13<br>Erdafitinib n=2<br>Other FGFR2 Inh. (not specified) n= 7
		2 FGFRi:<br>7 patients	Infigratinib/Derazantinib n=1<br>Pemigatinib/Derazantinib n=1<br>Pemigatinib/Futibatinib n=2<br>Derazantinib/Futibatinib n=1<br>Not specified n=2
		3 FGFRi:<br>1 patient	Pemigatinib/Futibatinib/Lirafugratinib n=1
FGFR2 non-fusion alteration	57	9 patients	
	Mutation: 41	1 FGFRi:<br>8 patients	Pemigatinib n=5<br>Derazantinib n=3
		2 FGFRi:<br>1 patient	Derazantinib/Futibatinib n=1
IDH<br>- IDH1<br>- IDH2	195<br>152<br>43	**50**<br>47<br>3	 Ivosidenib n=47<br>Enasidenib n=3
BRCA<br>- BRCA1<br>- BRCA2	67<br>29<br>38	15	Olaparib n=14<br>Olaparib+Pembrolizumab n=1

	No. of actionable GA	TT start overall cohort	Treatment
BRAF V600	29	14 (13 patients)	Dabrafenib+Trametinib n=8<br>Vemurafenib n=5<br>ERK-Inh. n=1
BRAF other	47		
- nonV600 mutation	41	1	Dabrafenib+Trametinib n=1
- amplification	3		
- unknown	3		
ERBB2 amplification	42	14	Trastuzumab+Pertuzumab n=5<br>Pertuzumab+Docetaxel n=1<br>Trastuzumab n=8
ERBB2 other	27		
- mutation	20	1	Trastuzumab n=1
- fusion	2	0	
- unknown	5	0	
Other/missing	21	24	Larotrectinib n=1 (NTRK amplification)<br>Erlotinib n=1 (not spec.)<br>Afatinib n=3 (NRG1 fusion)<br>Alpelisib n=1<br>Binimetinib+Capecitabine n=1 (not spec.)<br>Copanlisib+Gemcitabine+Cisplatin n=1 (not spec.)<br>Lapatinib+Trametinib n=2 (not spec.)<br>Olaparib n=2<br>Trabectidin+Olaparib n=1<br>Trastuzumab-Derutexan n=1<br>Rucaparin n=1<br>Pembrolizumab+Lenvatinib n=2<br>Pembrolizumab n=3<br>Ipilimumab+Nivolumab n=2<br>PDR001+INC280 n=1<br>Neratinib n=1
KRAS G12C	9	1	KRAS inhibitor n=1
MDM2 amplification	38	1	MDM2-inhibitor n=1
MET amplification	3	2	Tepotinib n=2
MTAP deletion	30	0	
PALB	16	0	
MSI	30	11	Pembrolizumab n=9<br>Nivolumab n=2
CPS	6	2	Pembrolizumab n=1<br>Nivolumab n=1

Abbreviations: *GA* genomic alterations, *FGFRi* FGFR inhibitor, *MSI* microsatellite instability; *CPS* combined positivie score

Fig. S1

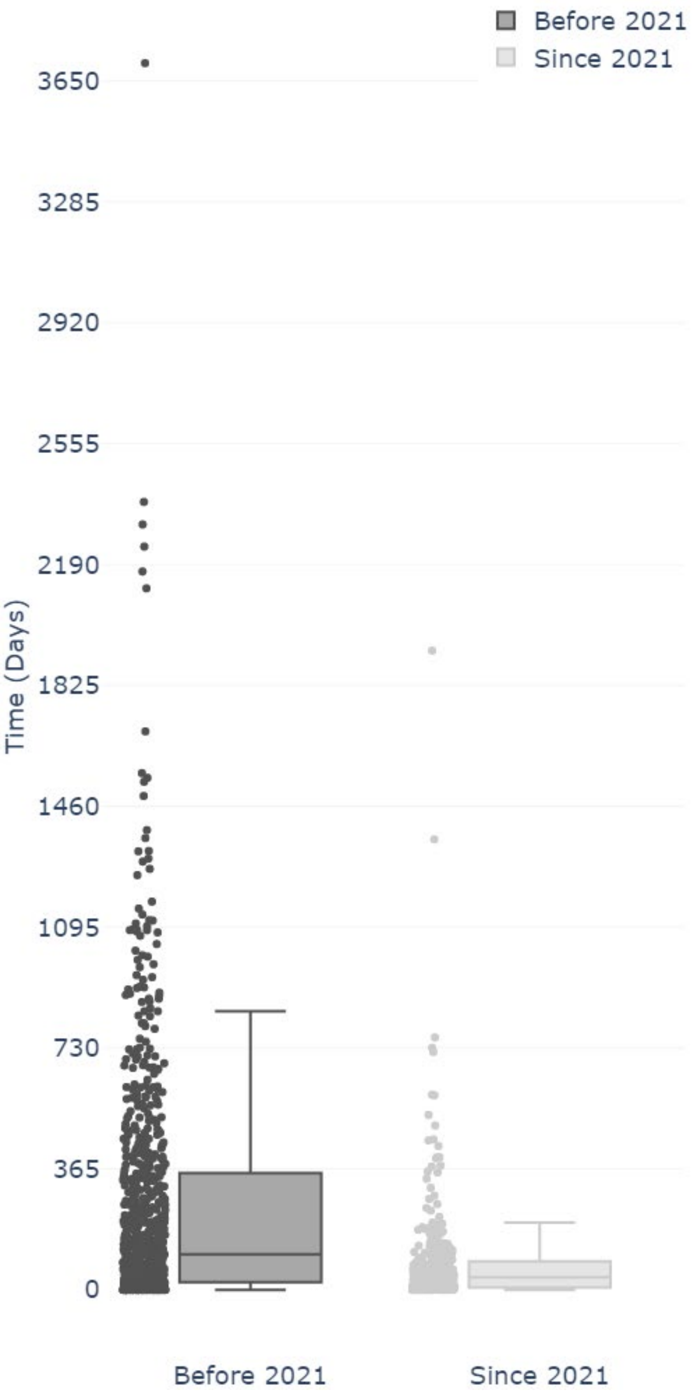

Time (days) between diagnosis of palliative stage and initial genomic analysis for patients receiving clinical genomic testing. Data were available for n = 1066 patients, with a median time of 65 days to genomic testing. Stratified into patients diagnosed before and after January 1, 2021 (before: n = 749; after: n = 317), time to testing was 107 days and 37 days, respectively (median). Data were not available for 293 patients. 162 tumor specimens with NGS data performed retrospectively for research purposes were excluded.

Fig. S2

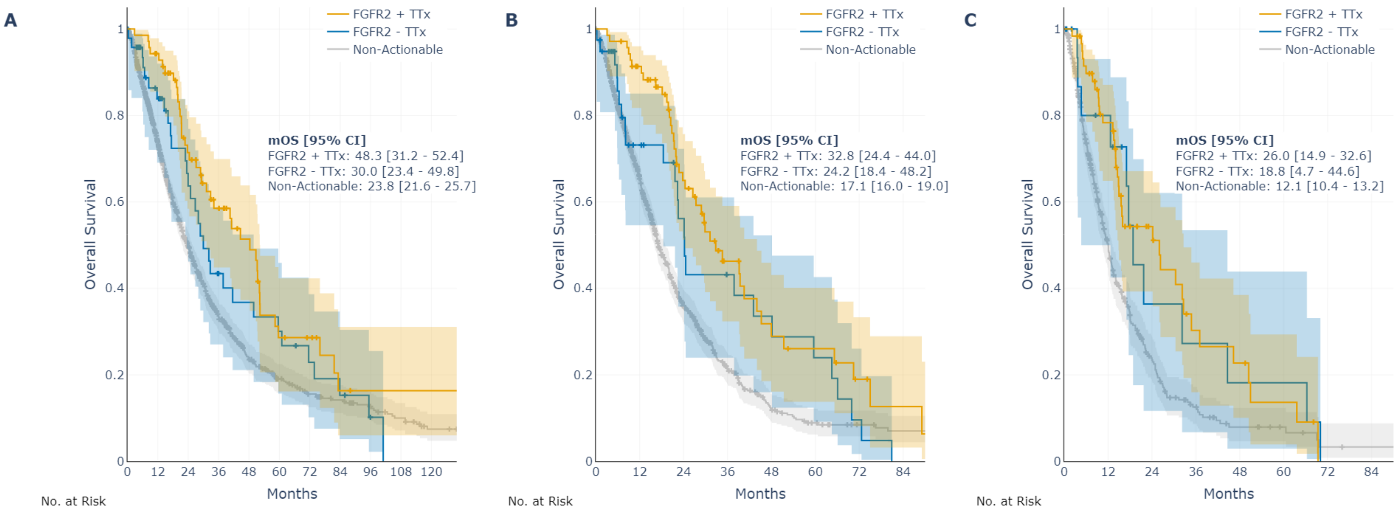

A

No. at Risk											
FGFR2 + TTx	70	64	43	30	22	12	10	5	4	4	4
FGFR2 - TTx	48	35	23	14	12	10	7	5	2	0	0
Non-Actionable	1036	655	370	196	106	69	50	41	31	23	13

B

No. at Risk								
FGFR2 + TTx	70	60	36	18	11	9	5	3
FGFR2 - TTx	39	22	14	10	8	6	3	0
Non-Actionable	872	441	190	82	38	22	14	11

C

No. at Risk								
FGFR2 + TTx	60	41	18	9	7	4	0	0
FGFR2 - TTx	18	12	5	4	3	3	0	0
Non-Actionable	398	149	53	23	11	7	3	2

OS for pts with an actionable FGFR2 fusions who received TTx, and for patients with and without the respective actionable GA. (A) OS for FGFR2 GA from diagnosis (+ TTx vs. - TTx: p = 0.192, HR = 0.70 [0.44 - 1.11]. (B) OS for FGFR2 fusions from the start of palliative treatment (+ TTx vs. - TTx: p = 0.132, HR = 0.70 [0.43 - 1.15]). (C) OS for FGFR2 GA from the start of second line treatment (+ TTx vs. - TTx: p = 0.975, HR = 0.88 [0.45 - 1.69]).

Abbreviations: *WT* wild-type, CI confidence interval, HR hazard ratio, mOS median overall survival, TTx targeted therapy, pts patients
