# Supplementary material for: Clinical practice and implications of biomarker testing in biliary tract cancer: An observational study
Source: JHEP Rep. 2025 Nov 4;8(1):101635. doi: 10.1016/j.jhepr.2025.101635 (PMC12890449; doi:10.1016/j.jhepr.2025.101635)
Supplement: Multimedia component 2 [file mmc2.docx]

**JHEP Reports**

**CTAT methods**

Tables for a “Complete, Transparent, Accurate and Timely account” (CTAT) are now mandatory for all revised submissions. The aim is to enhance the reproducibility of methods.

- Only include the parts relevant to your study
- Refer to the CTAT in the main text as ‘Supplementary CTAT Table’
- Do not add subheadings
- Add as many rows as needed to include all information
- Only include one item per row

**If the CTAT form is not relevant to your study, please outline the reasons why:**

|  |
| --- |

- 1. **Antibodies**

| **Name** | **Citation** | **Supplier** | **Cat no.** | **Clone no.** |
| --- | --- | --- | --- | --- |
|  |  |  |  |  |

- 1. **Cell lines**

| **Name** | **Citation** | **Supplier** | **Cat no.** | **Passage no.** | **Authentication test method** |
| --- | --- | --- | --- | --- | --- |
|  |  |  |  |  |  |

- 1. **Organisms**

| **Name** | **Citation** | **Supplier** | **Strain** | **Sex** | **Age** | **Overall n number** |
| --- | --- | --- | --- | --- | --- | --- |
|  |  |  |  |  |  |  |

- 1. **Sequence based reagents**

| **Name** | **Sequence** | **Supplier** |
| --- | --- | --- |
|  |  |  |

- 1. **Biological samples**

| **Description** | **Source** | **Identifier** |
| --- | --- | --- |
|  |  |  |

- 1. **Deposited data**

| **Name of repository** | **Identifier** | **Link** |
| --- | --- | --- |
|  |  |  |

- 1. **Software**

| **Software name** | **Manufacturer** | **Version** |
| --- | --- | --- |
| IBM SPSS Statistics software, version 28 (SPSSInc., Chicago, IL, USA) | IBM (SPSSInc., Chicago, IL, USA) | Version 28 |

- 1. **Other (*e.g*. drugs, proteins, vectors etc.)**

|  |  |  |
| --- | --- | --- |
|  |  |  |

- 1. **Please provide the details of the corresponding methods author for the manuscript:**

| **Prof. Arndt Vogel**  Clinician Scientist  Division of Gastroenterology and Hepatology, Toronto General Hospital  Medical Oncology, Princess Margaret Cancer Centre  Toronto General Hospital Research Institute  Schwartz Reisman Liver Research Centre Institute for Medical Science  200 Elizabeth Street, Office: 9 EB 236 Toronto, ON, M5G 2C4, Canada Email: Arndt.Vogel@uhn.ca; vogela@me.com  Web link: [Arndt Vogel, UHN](https://www.uhn.ca/PatientsFamilies/Search_Doctors/Pages/doctor_detail.aspx?doctor=1089) |
| --- |

**2.0 Please confirm for randomised controlled trials all versions of the clinical protocol are included in the submission. These will be published online as supplementary information.**

|  |
| --- |
